# Social Media Use and Well-being With Bipolar Disorder During the COVID-19 Pandemic: Path Analysis

**DOI:** 10.2196/39519

**Published:** 2022-08-18

**Authors:** Ariel Pollock Star, Yaacov G Bachner, Bar Cohen, Ophir Haglili, Norm O'Rourke

**Affiliations:** 1 Department of Epidemiology, Biostatistics and Community Health Sciences School of Public Health Ben-Gurion University of the Negev Be'er Sheva Israel; 2 Multidisciplinary Center for Research on Aging Faculty of Health Sciences Ben-Gurion University of the Negev Be'er Sheva Israel; 3 Goldman Medical School Faculty of Health Sciences Ben-Gurion University of the Negev Be'er Sheva Israel; 4 Department of Psychology Ben-Gurion University of the Negev Be'er Sheva Israel

**Keywords:** bipolar disorder, COVID-19, life satisfaction, loneliness, social media use, social media, Facebook, social support, mental health, mental illness, mental disorder, social media advertising, advertising, advertisement, mania, hypo/mania, manic, depressive, depression

## Abstract

**Background:**

Reliable and consistent social support is associated with the mental health and well-being of persons with severe mental illness, including bipolar disorder (BD). Yet the COVID-19 pandemic and associated social distancing measures (eg, shelter in place) reduced access to regular social contacts, while social media use (SMU) increased concomitantly. Little is currently known about associations between the well-being of adults with BD and different types of SMU (eg, passive and active).

**Objective:**

For this study, we had two goals. First, we report descriptive information regarding SMU by persons with BD during COVID-19 (all platforms). Specific to Facebook, we next developed and tested a hypothesized model to identify direct and indirect associations between BD symptoms, social support, loneliness, life satisfaction, and SMU. Responses were collected during the global spread of the Delta variant and prior/concurrent with the Omicron variant, 20 months after the World Health Organization declared COVID-19 a global pandemic.

**Methods:**

Over 8 weeks, we obtained responses from an international sample of 102 adults with BD using the Qualtrics online platform. Most had previously participated in the BADAS (Bipolar Affective Disorders and older Adults) Study (n=89, 87.3%); the remainder were recruited specifically for this research (n=13, 2.7%). The subsamples did not differ in age (*t*_100_=1.64; *P*=.10), gender (*χ*^2^_2_=0.2; *P*=.90), socioeconomic status (*χ*^2^_6_=9.9; *P*=.13), or time since BD diagnosis (*t*_97_=1.27; *P*=.21). Both were recruited using social media advertising micro-targeted to adults with BD. On average, participants were 53.96 (SD 13.22, range 20-77) years of age, they had completed 15.4 (SD 4.28) years of education, and were diagnosed with BD 19.6 (SD 10.31) years ago. Path analyses were performed to develop and test our hypothesized model.

**Results:**

Almost all participants (n=95, 93.1%) reported having both Facebook and LinkedIn accounts; 91.2% (n=93) reported regular use of either or both. During the pandemic, most (n=62, 60.8%) reported accessing social media several times a day; 36.3% (n=37) reported using social media more often since the emergence of COVID-19. Specific to Facebook, the model we hypothesized differed somewhat from what emerged. The resulting model suggests that symptoms of depression predict loneliness and, inversely, social support and life satisfaction. Social support predicts social Facebook use, whereas passive Facebook use predicts life satisfaction. Symptoms of depression emerged as indirect predictors of SMU via social support.

**Conclusions:**

Our findings suggest that the operational definition of passive-active SMU requires further analysis and refinement. In contrast to theory, passive Facebook use appears positively associated with well-being among certain populations. Longitudinal data collection over multiple points is required to identify associations between BD symptoms, SMU, and well-being over time.

## Introduction

### Social Support With Bipolar Disorder

Well-being with bipolar disorder (BD) has many facets; for most, this entails routine and consistency, including regular social contact and support [1]. The importance of social support for adults with BD is well documented. It has been shown, for instance, that greater contact with friends and family fosters well-being with BD [2], and those with less social support report more symptoms of mania [3]. The perceived absence of social support is associated with the recurrence of BD mood episodes [4] and slower time to recovery [5].

Isolation and disruption in routine caused by the COVID-19 pandemic have been especially difficult for those with mental health conditions. For instance, one Israeli study showed that those who reported being most lonely during the pandemic were 82% more likely to struggle with depression and anxiety [6]. A similar US study reported that greater loneliness was associated with increased symptoms of depression and suicidal ideation [7].

Initial research on the psychological effects of COVID-19 early in the pandemic reported no significant effects on persons with mental health conditions compared to prepandemic symptoms [8,9]. Some lifestyle changes caused by the COVID-19 pandemic restrictions (eg, lowered access to social support) were less pronounced for persons with BD than healthy controls [10]. This may be due to fewer social contacts at baseline for those with BD (ie, smaller social networks or family estrangement). However, reduced access to social support, along with disruptions to routine, lower income, and unemployment, have a longer-lasting impact on those with BD, leading to longer-term disruptions [11]. This is reflected in recent research [11-13]. In a study from Australia, those with mood disorders (depressive disorder or BD) reported elevated levels of psychological distress (ie, stress, anxiety, or depression) when compared to those free of psychopathology [12].

### Social Media Use and Mood Disorders

In the absence of interpersonal contact, many turned to various social media [14]. With an estimated 4.6 billion global users [15], social media (eg, Facebook, Instagram, Twitter, or TikTok) have become platforms, and in some cases replacements, for in-person community building and support [16]. This has been particularly true during the global COVID-19 pandemic [17,18] when in-person social support ceased to be an option for many, particularly those living alone. Yet our understanding of the effects of social media use (SMU) on mental health remains incomplete despite the omnipresence of social media in modern life.

Excessive SMU may well cause or exacerbate symptoms of depression. Among adolescent and young adults, associations between depressive symptoms and SMU are well established [19-21]. Research on persons with chronic depression suggests that an increase in SMU exacerbates symptoms [22]. Alternatively, depression may predict excessive SMU [23], as those with severe chronic depression are often socially isolated and rely on online interactions [24]. Different facets of SMU may play differing roles, as active versus passive use patterns appear to have opposing associations with social comparison and, in turn, depressive symptoms [25].

Most SMU and mental health research to date has focused on depression and anxiety [26]. The relationship between SMU and other mental health conditions is even less well understood. BD is an especially stigmatized mental health condition as reflected online; tweets relating to BD were found to be more stigmatizing than those pertaining to other mental health conditions [27]. This may create a complex online experience for those with BD. However, research also points to the potential for social media to confer peer support to those with BD, either as a supplement or alternative to in-person support. Qualitative research findings suggest that those with severe mental illness, including BD, seek out opportunities to connect with peers online [28] and that peer support occurs naturally among those who share their experiences online [29]. In intervention research, intentional weight loss was fostered by online support and interaction [30].

### Passive and Active Social Media Use

Research conducted with general adult samples underscores that SMU is not a singular behavior [16]; instead, there are various ways in which social media are used, with differential effects on mental health and well-being [31]. Active SMU entails direct engagement with social media, such as posting comments or commenting on posts. Active use includes sharing pictures, opinions, or interests to communicate or connect with friends and family. In contrast, passive SMU entails consuming online information without posting or commenting (eg, scrolling news feeds and viewing posts). This behavior allows the user to observe other people while maintaining relative anonymity.

There is no consensus regarding associations between different patterns of SMU and well-being. Multiple studies suggest that active SMU is negatively associated with depressive symptoms [32] and perceived loneliness, and that this relationship is mediated by social support [33,34]. Yet other studies suggest that passive SMU is negatively associated with well-being because it encourages social comparison and feelings of envy [35], and is associated with a depressed mood [36,37]. However, the active-positive and passive-negative dichotomy is not universally accepted. One study performed during the COVID-19 pandemic initially found a positive association between active SMU and meaning in life, yet in a replication study, the opposite was found as well as an association between active SMU and emotional loneliness [38]. There is also evidence that passive SMU is associated with positive well-being such as life satisfaction [39].

This lack of consensus regarding the effects of active and passive SMU may be due in part to different definitions of terms and scales. A meta-analysis found that, of 40 studies of active and passive SMU and well-being, there were 36 different operational definitions of active and passive SMU [40]. Existing scales do not fully differentiate between different nuances of active and passive SMU (eg, public and private use or social and nonsocial use) and are not universally applicable to the range of social media platforms that exist today [35,40]. The effects of social media on the well-being of adults with severe mental illness are unclear. Additionally, the role of social media in daily life may have become even more prominent as a result of the COVID-19 pandemic.

**Figure 1:**
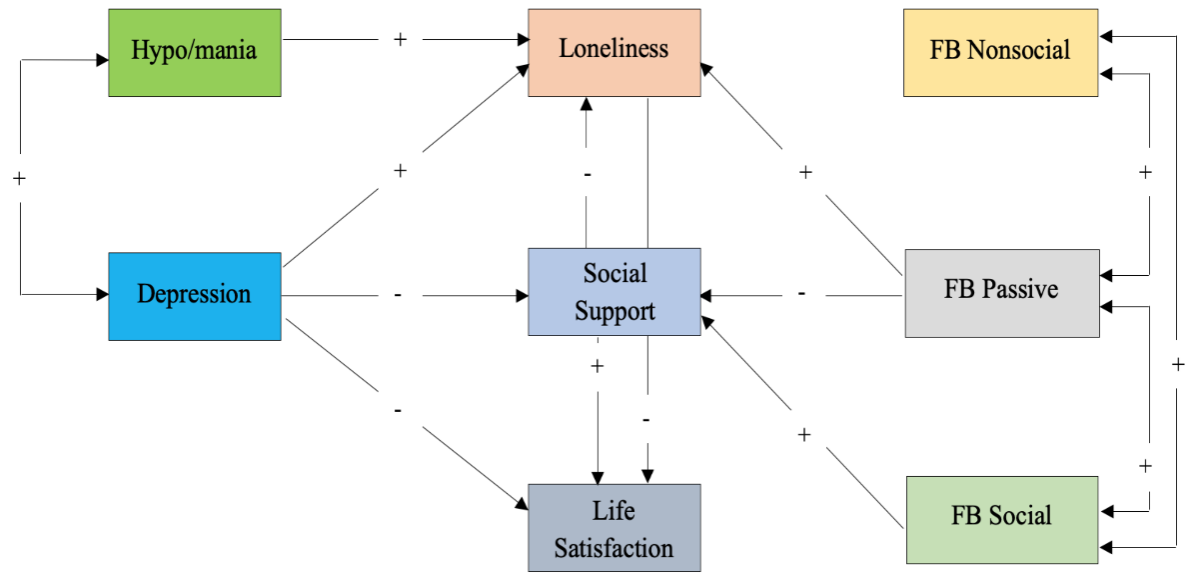
Statistically significant associations depicted as paths (directional arrows) or correlated associations (double-headed arrows). Positive (+) and negative (-) directional associations hypothesized a priori. FB: Facebook.

The aims of this study were twofold. First, we set out to describe general patterns of SMU by adults with BD during COVID-19 (all platforms). Specific to Facebook, we next tested a hypothesized model to identify direct and indirect associations between BD symptoms, social support, loneliness, life satisfaction, and SMU (passive, active social, and active nonsocial; see Figure 1).

In keeping with previously published research, we hypothesized that:

- Social Facebook use would predict social support
- Passive Facebook use would predict both loneliness and lower social support
- Depressive symptoms would predict lower life satisfaction
- Depressive symptoms would predict loneliness and lower social support
- Symptoms of hypo/mania would predict loneliness

## Methods

### Online Recruitment and Data Collection

Most (n=89, 87.3%) of the 102 participants previously took part in the Bipolar Affective Disorders and Older Adults (BADAS) study [41]; the remainder were recruited specifically for this study. Subsamples did not differ in age (*t*_100_=1.64; *P*=.10), gender (*χ*^2^_2_=0.2; *P*=.90), socioeconomic status (*χ*^2^_6_=9.9; *P*=.13), or country of residence (*χ*^2^_3_=6.0; *P*=.11). They also did not differ in symptoms of depression (*t*_100_=.93; *P*=.36), hypo/mania (*t*_100_=0.95; *P*=.35), or time since BD diagnosis (*t*_97_=1.27; *P*=.21). Both subsamples were recruited using microtargeted Facebook advertising. This method has been used effectively to recruit clinical samples from low prevalence populations, including BD [42].

BADAS participants who authorized future contact provided their email addresses [41,43]. They were sent up to three personalized notices requesting their participation in this study. A first email was sent in early November 2021, a reminder was sent 1 week later (if there was no response to the first), and 3 weeks thereafter (if there was no response to the first or second).

Concurrently, a Facebook page was established, and microtargeted advertisements were sent to prospective new participants using the A/B test method (ie, two versions of the same advertisement compared). The more successful was then used in the third round of advertising. Prospective participants were ≥18 years of age, could read and write English, and were members of ≥1 online BD advocacy or support group. Responses were obtained during the global spread of the Delta variant and prior/concurrent with the Omicron variant, 20 months after the World Health Organization declared COVID-19 a global pandemic.

Both BADAS and newly recruited participants were directed to an online questionnaire hosted on the Qualtrics platform. Participants were initially asked their age in years and, later, their date of birth in the demographics questionnaire to corroborate candid responding. To facilitate data collection, participants could enter a lottery to win a single US $500 prize (ie, an Amazon gift card).

### Ethics Approval

Ethics approval was received from the Institutional Review Board at Ben-Gurion University of the Negev, Be’er Sheva, Israel (#2157-2). By clicking to proceed, respondents indicated consent to participate as stated on the study splash page. They were not required to provide identifying information aside from an email address if they wished to receive a study summary or participate in the lottery.

### Instruments

The Bipolar Disorder Symptom Scale (BDS_x_) was developed to measure symptoms of both depression and hypo/mania (hypomania + mania = hypo/mania: continuum where the point of transition is not immediately apparent) [43-45]. Respondents indicate the extent to which each of the 20 items describes how they feel at this moment, on a Likert scale ranging from *not at all* (0) to *a lot* (2). Internal consistency of BDS_x_ responses by BD outpatients was reported as α=.90 for the depression subscale (cognitive + somatic symptoms) [46] but lower for the hypo/mania subscale at α=.76 (affrontive + elation and loss of insight) [47]. This difference may be due to the low frequency of hypo/manic versus depressive symptoms [48].

Concurrent validity of BDS_x_ responses by BD outpatients has been demonstrated relative to the self-reported Hamilton Rating Scale for Depression and the Altman Self-Rating Mania Scale [47]. Similarly, sensitivity and specificity are high for the BDS_x_ depression subscale at 88% and 76%, respectively [46]. Sensitivity is lower at 57% for the BDS_x_ hypo/mania subscale (90% specificity), but sensitivity is higher than for the Altman scale (43%) [46].

The Satisfaction with Life Scale (SLS) [49] measures perceived quality of life based on person-specific criteria [50]. The SLS is composed of five questions (eg, “The conditions of my life are excellent”) with response alternatives ranging from *strongly disagree* (1) to *strongly agree* (7). Higher totals are suggestive of greater life satisfaction [51]. Test-retest reliability over 1-month has been reported as *r*=0.84 [52]. Concurrent validity of SLS responses has been demonstrated relative to the Fordyce Global Scale (*r*=0.82) [34]. Among adults with BD, internal consistency has been reported as α=.89 [53].

The eight-item UCLA Loneliness Scale (ULS-8) is a brief measure developed to study relationships between loneliness and health-related behavior [54]. Responses are reported along a Likert scale ranging from *I never feel this way* (0) to *I often feel this way* (3). Internal consistency of ULS-8 responses is high (α=.84) [55].

The Multidimensional Scale of Perceived Social Support is a 12-item measure of subjective social support from partners, family, and friends [56]. Responses are reported on a Likert scale ranging from *very strongly disagree* (1) to *very strongly agree* (7). High internal consistency was reported in scale development (α=.88 [56]) and subsequent research (.83<α<.93 [57,58]).

The Passive and Active Facebook Use Measure (PAUM) was developed to distinguish different types of Facebook use [59]. Exploratory factor analyses suggest three distinct patterns: active social, active nonsocial, and passive. Active social use pertains to direct engagement (eg, chatting or commenting to Facebook posts), whereas active nonsocial use does not entail direct interaction with others (eg, post videos or tag photos), and passive use is limited to viewing photos and checking the status of others (ie, no engagement). Internal consistency across subscales ranges from adequate to good (.70<α<.81 [59,60]).

We created a self-report questionnaire to collect demographic information. Participants indicated their country of residence (drop-down menu), number of years of education, work or occupation, employment status, and current relationship status. They were asked their gender, ethnicity, if they had been diagnosed with BD by a clinician (eg, psychiatrist), BD subtype if known, and date of BD diagnosis (month, year).

### Statistical Methods

Path analysis was performed for this study as a three-step process [61]. A hypothesized model was first tested, nonsignificant paths were deleted, and statistically significant paths not initially hypothesized were added if supported by existing research or theory (see Figure 1).

Path analysis is an extension of multiple linear regression with three significant advantages. Path analysis allows us to simultaneously predict one or more dependent variables (touched by arrowheads in path models). Arrows pointing from independent to dependent variables represent significant prediction (ie, critical ratio values >|1.96|, *P*<.05). Path analysis is a multivariate statistical procedure meaning that all significant paths emerged concurrently (ie, over and above other statistically significant results).

Path models allow us to identify both direct and indirect predictors; indirect prediction occurs via other variables (ie, ≥2 pathways between variables). In complex or more nuanced models, variables can have both direct and indirect effects on dependent variables; indirect effects can be of equal or greater magnitude than direct effects (total effects = direct + indirect effects).

Computing path analyses with structural equation modeling software allows us to obtain goodness of fit information for the overall model. Good model fit is required to interpret individual results [62]. In accord with convention, we report three goodness of fit indexes to assess overall model fit: an incremental (comparative fit index [CFI]), an absolute (standardized root mean residual [SRMR]), and a parsimonious fit index (root mean square error of approximation [RMSEA]). Ideal SRMR and RMSEA values are less than 0.055, whereas ideal CFI values are greater than 0.95 [61]. Descriptive and comparative analyses were performed using SPSS v28 (IBM Corp), and path analyses were performed using AMOS v28 and maximum likelihood estimation [62].

## Results

### Descriptive Features

We recruited 102 participants over 8 weeks who were 53.96 (SD 13.22; range 20-77) years of age on average, had completed 15.4 (SD 9.88) years of education, and were diagnosed with BD 19.61 (SD 10.29; range 1-58) years ago. Most participants were women (n=69, 67.6%) and currently married, cohabitating, or partnered (n=56, 54.9%); 27 were single, 11 separated or divorced, and 4 widowed. Most lived in North America (Canada: n=45, United States: n=15), Western Europe (eg, United Kingdom: n=18, Ireland: n=10), South Africa (n=4), and Australia (n=4) or New Zealand (n=3). Table 1 reports descriptive statistics and the psychometric information for scale responses (eg, internal consistency). Responses are largely within normal limits (limited skewness and kurtosis) with adequate to ideal internal consistency for almost all study measures (see Table 1).

**Table 1:** Descriptive features and psychometric statistics for study variables (N=102).

	Mean (SD)	Range	Skewness	Kurtosis	Cronbach α^a^
Age (years)	53.96 (13.22)	20-77	–0.40	–0.73	—^b^
Education (years)	15.40 (4.28)	2-25	0.05	1.28	—
Duration BD D_x_^c^ (years)	19.60 (10.31)	1-58	1.17	1.57	—
BDS_x_^d^: depression	9.94 (5.69)	0-22	–0.01	–0.91	.89
BDS_x_: hypo/mania	3.72 (3.39)	0-13	0.93	–0.11	.78
Life satisfaction	16.69 (7.50)	5-35	0.49	–0.60	.89
Loneliness^e^	20.52 (5.52)	8-32	–0.15	–0.91	.83
Social support^f^	53.94 (18.53)	12-84	–0.44	–0.43	.93
Facebook^g^ passive	6.84 (3.25)	0-13	–0.47	–0.13	.67
Facebook social	7.76 (4.39)	0-18	–0.03	–0.46	.83
Facebook nonsocial	2.22 (2.62)	0-12	1.47	2.12	.70

^a^Internal consistency of scale responses as measured by Cronbach alpha.

^b^Not applicable.

^c^BD D_x_: bipolar disorder diagnosis.

^d^BDS_x_: Bipolar Disorder Symptom Scale.

^e^Loneliness: UCLA Loneliness Scale.

^f^Social support: Multidimensional Scale of Perceived Social Support.

^g^Facebook: passive and active Facebook use.

### Social Media Use During COVID-19

All participants reported living under government-regulated social distancing or shelter-in-place restrictions, either prior or concurrent to completing the study questionnaire. When asked, 60.8% (n=62) of the 102 participants reported using social media multiple times a day, with Facebook and LinkedIn the most commonly used platforms: 93.1% (n=95) reported accounts on either or both platforms and 91.2% (n=93) reported regular use of either or both. More than one-third (n=37, 36.3%) reported using social media more since the start of the COVID-19 pandemic.

### BD Symptoms, Life Satisfaction, and Facebook Use During COVID-19

We performed path analyses to test our hypothesized model of Facebook use (see Figure 1). Symptoms of depression were assumed to predict loneliness, lower social support, and lower life satisfaction. Use of Facebook was assumed to indirectly predict life satisfaction via social support. Our sample of 102 participants is not large but sufficient to detect medium to large effect sizes with 7 independent variables (where α=.05, *d*=0.80) [63].

**Figure 2:**
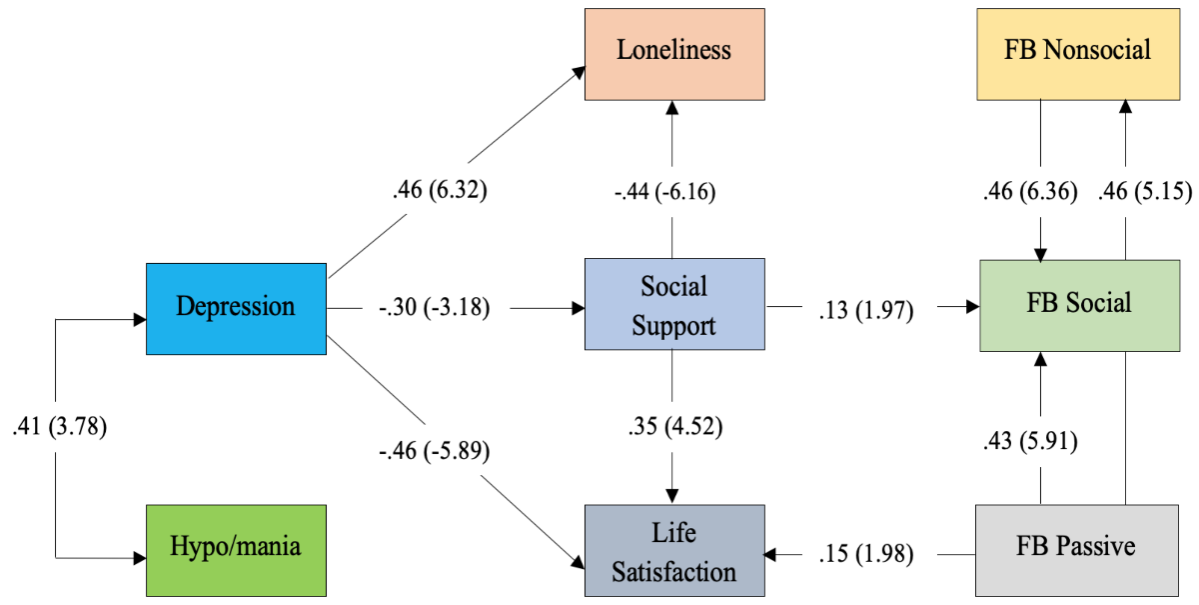
Direct and indirect predictors of Facebook use and life satisfaction with bipolar disorder during COVID-19. Parameters are expressed as maximum likelihood estimates (standardized solution). Parenthetical numbers indicate significance levels (ie, Critical Ratio [CR] values >|1.96|, *P*<.05; CR>|2.58|, *P*<.01). FB: Facebook.

A somewhat different model emerged (see Figure 2). Goodness of fit for this path model was within optimal parameters for two of three statistics examined (*χ*^2^_17_=15.7; *P*=.55). That is, the comparative fit index (CFI>.95; CFI=.99) and the root mean square error of approximation (RMSEA<0.055, RMSEA=0.001; 0<RMSEA 90% confidence limits<0.083) were both within ideal limits. The SRMR was within adequate parameters (SRMR<0.055; SRMR=0.06).

Consistent with previous research [64], symptoms of depression and hypo/mania are positively correlated; only the former, however, significantly predicts loneliness (β=.46; *P<*.001), social support (β=–.30; *P*=.001), and life satisfaction (β=–.46; *P*<.001). Passive Facebook use predicts life satisfaction (β=.15; *P=*.048), and social support predicts social Facebook use (β=.13; *P*=.048), not the reverse as originally predicted. Fully 45% of variance in life satisfaction with BD is explained by this model (*R*^2^=0.45; *P*<.001; see Table 2).

Depressive symptoms appear to have a direct and indirect effect on both loneliness and life satisfaction via social support, and depressive symptoms have a small indirect effect on social Facebook use. We assumed that the various aspects of Facebook use would predict social support and life satisfaction with BD; however, associations appear bidirectional. Facebook use is both a predictor of life satisfaction and predicted by social support.

**Table 2:** Direct and indirect predictors of life satisfaction and Facebook use by adults with bipolar disorder.^a^

		Depression		FB^b^ passive	FB nonsocial		Support
**FB nonsocial**							
	Direct	—^c^	0.46		—	—	
	Indirect	—	0.00		—	—	
	Total	—	0.46		—	—	
**Social support**							
	Direct	–0.30	—		—	—	
	Indirect	0.00	—		—	—	
	Total	–0.30	—		—	—	
**Loneliness**							
	Direct	0.46	—		—	–0.44	
	Indirect	0.13	—		—	0.00	
	Total	0.59	—		—	–0.44	
**Life satisfaction**							
	Direct	–0.46	0.15		—	0.35	
	Indirect	–0.11	0.00		—	0.00	
	Total	–0.56	0.15		—	0.35	
**FB social**							
	Direct	0.00	0.43		0.46	0.13	
	Indirect	–0.04	0.21		0.00	0.00	
	Total	–0.04	0.64		0.46	0.13	

^a^Numbers represent percentages of variance explained by each variable (direct variance=path in model).

^b^FB: Facebook.

^c^Not applicable.

## Discussion

### Study Hypotheses

As expected, a significant association emerged between social support and Facebook use; however, the direction of this association was opposite than expected, with social support predicting social Facebook use, not the reverse. Additionally, contrary to our hypothesized model, passive Facebook use predicted life satisfaction. These findings suggest bidirectional associations between different facets of SMU and well-being with BD during the COVID-19 pandemic.

As predicted, depressive symptoms were negatively associated with both social support and life satisfaction, and positively associated with loneliness. Though symptoms of hypo/mania and depression were correlated, no direct or indirect associations emerged between hypo/mania and measures of well-being or SMU.

### Comparison With Previous Research

These results are consistent with prior research indicating a positive association between symptoms of depression and hypo/mania [65]. Consistent with existing research, our findings confirm that Facebook use is not a singular behavior but multifaceted [66], with differential effects upon social support and well-being [25]. Our finding that social support predicts active social Facebook use, but not other forms of Facebook use, suggests that those with stronger feelings of in-person social support may be more inclined to use social media to maintain connections online. This positive association may be understood in terms of the outsize role that social media played in providing a substitute for in-person social support during the COVID-19 pandemic; this finding needs to be corroborated with other populations with and without mental illness.

Our result suggesting a positive association between passive Facebook use and life satisfaction is contrary to most prior research with general adult samples [60]. However, this finding is consistent with other research indicating a more complex relationship between SMU types and well-being [44]. The association between passive Facebook use and life satisfaction may indicate that for certain populations, such as older adults or persons with BD, passive behavior is not indicative of social comparison or lack of self-confidence but rather of neutral or positive character traits such as contemplativeness or sense of self. Note that passive and active SMU are defined differently by different scales, thus leading to ambiguity regarding how these facets of SMU are related to well-being. The role of active nonsocial Facebook use on well-being requires further research; no association with measures of well-being emerged for this study. This may be due to the lower levels of active nonsocial Facebook use in our sample.

SMU has become central to daily life and functioning [67]; over the course of the COVID-19 pandemic, social media became even more deeply embedded in daily life, integral for social networking when opportunities for in-person social interaction were limited or nonexistent. It remains to be seen whether SMU receded once the pandemic began to wane or whether digital relationships continued to take precedence over in-person interactions. There exists a need for both more universal measurement across social media platforms and more comprehensive measurement of all facets of active and passive use.

These questions remain even more pressing for those with severe mental health conditions such as BD. The timing of this study allowed us to explore relationships between SMU and indexes of well-being during a time when in-person social interaction was limited for all, not only for adults with mental health conditions who tend to self-isolate. This provided an opportunity to begin to explore the role of isolation in SMU and well-being. The findings of this study provide preliminary support for the assertion that those with mental health conditions and limited in-person social networks may benefit from certain types of SMU. As indicated by our findings, even passive SMU is suggestive of life satisfaction in this sample of adults with BD.

### Limitations and Future Research

This study had several limitations. Both BADAS participants and newly recruited participants were recruited using Facebook. As discussed elsewhere, persons with BD recruited via social media are more symptomatic than psychiatric outpatients [46] and may not be representative of the population of persons with BD. Additionally, persons recruited via social media are likely more regular users than the general population, and participants recruited via Facebook self-reported BD diagnoses (date and BD subtype); this information was not corroborated by chart review or structured clinical interview.

Existing instruments measuring SMU are currently limited [68]. Though developed for use with general adult samples, the psychometric properties of PAUM responses suggest good internal consistency and concurrent validity with adults with BD. Especially with psychiatric samples, future research is needed examining problematic SMU (eg, addiction) [69]. Though the PAUM appears to be the most widely used measure of SMU, it measures active and passive Facebook use only. Future research is needed examining the effects of other social media platforms on mental health and well-being [35].

The results of this study need to be replicated with larger samples recruited by more traditional methods (eg, psychiatric outpatients). A sample of 102 participants was sufficient to compute a path model with up to seven independent variables; larger samples are required to identify small effect sizes. Longitudinal research in particular is necessary to understand variability in social support and SMU, especially in relation to change in BD symptoms over time.

### Conclusions and Summary

The majority of existing research on SMU and well-being has examined young healthy adults. However, social media is today used by most of the world and pervades every population irrespective of age, socioeconomic status, and health. Due to the proliferation of platforms and social media options, different populations may use and relate to social media in distinct ways. To understand the effect of these distinct use patterns and their effects on well-being, it is necessary to study use patterns across diverse populations over time.

The aim of this study was to describe general patterns of SMU by adults with BD during COVID-19 across social media platforms and then to develop and test a hypothesized model specific to Facebook use to identify direct and indirect associations between BD symptoms, social support, loneliness, life satisfaction, and SMU. Contrary to our hypotheses that the patterns of association would conform with the research, indicating active-positive and passive-negative associations with well-being, we found distinct patterns of association.

Our results suggest that adults with BD may use and relate to social media differently than general adult samples. There is a need for further tool development to measure and compare different types of SMU [37] and for longitudinal research examining associations between SMU types and well-being of adults with BD and other forms of severe mental illness (eg, schizophrenia).

## Abbreviations

BADAS: Bipolar Affective Disorders and Older Adults
BD: bipolar disorder
BDSx: Bipolar Disorder Symptom Scale
CFI: comparative fit index
PAUM: Passive and Active Facebook Use Measure
RMSEA: root mean square error of approximation
SLS: Satisfaction with Life Scale
SMU: social media use
SRMR: standardized root mean residual
ULS-8: eight-item UCLA Loneliness Scale

## References

[1] Poole R, Smith D, Simpson S (2015). How patients contribute to an online psychoeducation forum for bipolar disorder: a virtual participant observation study. JMIR Ment Health.

[2] Gutiérrez-Rojas L, Gurpegui M, Ayuso-Mateos JL, Gutiérrez-Ariza JA, Ruiz-Veguilla M, Jurado D (2008). Quality of life in bipolar disorder patients: a comparison with a general population sample. Bipolar Disord.

[3] Dunne L, Perich T, Meade T (2019). The relationship between social support and personal recovery in bipolar disorder. Psychiatr Rehabil J.

[4] Cohen AN, Hammen C, Henry RM, Daley SE (2004). Effects of stress and social support on recurrence in bipolar disorder. J Affect Disord.

[5] Johnson SL, Winett CA, Meyer B, Greenhouse WJ, Miller I (1999). Social support and the course of bipolar disorder. J Abnorm Psychol.

[6] Palgi Y, Shrira A, Ring L, Bodner E, Avidor S, Bergman Y, Cohen-Fridel S, Keisari S, Hoffman Y (2020). The loneliness pandemic: loneliness and other concomitants of depression, anxiety and their comorbidity during the COVID-19 outbreak. J Affect Disord.

[7] Killgore WD, Cloonan SA, Taylor EC, Dailey NS (2020). Loneliness: a signature mental health concern in the era of COVID-19. Psychiatry Res.

[8] Lewis KJS, Gordon-Smith K, Saunders KEA, Dolman C, South M, Geddes J, Craddock N, Di Florio A, Jones I, Jones L (2022). Mental health prior to and during the COVID-19 pandemic in individuals with bipolar disorder: insights from prospective longitudinal data. Bipolar Disord.

[9] Orhan M, Korten N, Paans N, de Walle B, Kupka R, van Oppen P, Kok A, Sonnenberg C, Schouws S, Dols A (2021). Psychiatric symptoms during the COVID-19 outbreak in older adults with bipolar disorder. Int J Geriatr Psychiatry.

[10] Dalkner N, Ratzenhofer M, Fleischmann E, Fellendorf FT, Bengesser S, Birner A, Maget A, Großschädl K, Lenger M, Platzer M, Queissner R, Schönthaler E, Tmava-Berisha A, Berndt C, Martini J, Bauer M, Sperling JD, Vinberg M, Reininghaus EZ (2022). Psychological and behavioral response on the COVID-19 pandemic in individuals with bipolar disorder: a multicenter study. Psychiatry Res.

[11] Yocum AK, Zhai Y, McInnis MG, Han P (2021). Covid-19 pandemic and lockdown impacts: A description in a longitudinal study of bipolar disorder. Journal of Affective Disorders.

[12] Van Rheenen TE, Meyer D, Neill E, Phillipou A, Tan EJ, Toh WL, Rossell SL (2020). Mental health status of individuals with a mood-disorder during the COVID-19 pandemic in Australia: Initial results from the COLLATE project. Journal of Affective Disorders.

[13] Karantonis JA, Rossell SL, Berk M, Van Rheenen TE (2021). The mental health and lifestyle impacts of COVID-19 on bipolar disorder. Journal of Affective Disorders.

[14] Tuck AB, Thompson RJ (2021). Social networking site use during the COVID-19 pandemic and its associations with social and emotional well-being in college students: survey study. JMIR Form Res.

[15] (2022). Global social media statistics. DataReportal.

[16] Twenge JM, Spitzberg BH, Campbell WK (2019). Less in-person social interaction with peers among U.S. adolescents in the 21st century and links to loneliness. Journal of Social and Personal Relationships.

[17] Singh S, Dixit A, Joshi G (2020). “Is compulsive social media use amid COVID-19 pandemic addictive behavior or coping mechanism?. Asian Journal of Psychiatry.

[18] Boursier V, Gioia F, Musetti A, Schimmenti A (2020). Facing loneliness and anxiety during the COVID-19 isolation: the role of excessive social media use in a sample of Italian adults. Front. Psychiatry.

[19] Lin LY, Sidani JE, Shensa A, Radovic A, Miller E, Colditz JB, Hoffman BL, Giles LM, Primack BA (2016). Association between social media use and depression among U.S. young adults. Depress Anxiety.

[20] Primack BA, Shensa A, Escobar-Viera CG, Barrett EL, Sidani JE, Colditz JB, James AE (2017). Use of multiple social media platforms and symptoms of depression and anxiety: A nationally-representative study among U.S. young adults. Computers in Human Behavior.

[21] Shensa A, Sidani JE, Dew MA, Escobar-Viera CG, Primack BA (2018). Social media use and depression and anxiety symptoms: a cluster analysis. am j health behav.

[22] Taylor-Jackson J, Moustafa A (2021). The relationships between social media use and factors relating to depression. Nat Depression.

[23] Raudsepp L, Kais K (2019). Longitudinal associations between problematic social media use and depressive symptoms in adolescent girls. Preventive Medicine Reports.

[24] Cole DA, Nick EA, Varga G, Smith D, Zelkowitz RL, Ford MA, Lédeczi ? (2019). Are aspects of Twitter use associated with reduced depressive symptoms? The moderating role of in-person social support. Cyberpsychology, Behavior, and Social Networking.

[25] Nisar TM, Prabhakar G, Ilavarasan PV, Baabdullah AM (2019). Facebook usage and mental health: An empirical study of role of non-directional social comparisons in the UK. International Journal of Information Management.

[26] Arias-de la Torre J, Puigdomenech E, García X, Valderas JM, Eiroa-Orosa FJ, Fernández-Villa T, Molina AJ, Martín V, Serrano-Blanco A, Alonso J, Espallargues M (2020). Relationship between depression and the use of mobile technologies and social media among adolescents: umbrella review. J Med Internet Res.

[27] Budenz A, Klassen A, Purtle J, Yom Tov E, Yudell M, Massey P (2020). Mental illness and bipolar disorder on Twitter: implications for stigma and social support. J Ment Health.

[28] Naslund JA, Aschbrenner KA, Marsch LA, Bartels SJ (2016). The future of mental health care: peer-to-peer support and social media. Epidemiol Psychiatr Sci.

[29] Naslund JA, Grande SW, Aschbrenner KA, Elwyn G (2014). Naturally occurring peer support through social media: the experiences of individuals with severe mental illness using YouTube. PLoS One.

[30] Naslund JA, Aschbrenner KA, Marsch LA, McHugo GJ, Bartels SJ (2017). Facebook for supporting a lifestyle intervention for people with major depressive disorder, bipolar disorder, and schizophrenia: an exploratory study. Psychiatr Q.

[31] Fioravanti G, Casale S (2020). The active and passive use of Facebook: measurement and association with Facebook addiction. J Psychopathol.

[32] Escobar-Viera CG, Shensa A, Bowman ND, Sidani JE, Knight J, James AE, Primack BA (2018). Passive and active social media use and depressive symptoms among United States adults. Cyberpsychol Behav Soc Netw.

[33] Lin S, Liu D, Niu G, Longobardi C (2020). Active social network sites use and loneliness: the mediating role of social support and self-esteem. Curr Psychol.

[34] Zhang K, Kim K, Silverstein NM, Song Q, Burr JA (2021). Social media communication and loneliness among older adults: the mediating roles of social support and social contact. Gerontologist.

[35] Masciantonio A, Bourguignon D, Bouchat P, Balty M, Rimé B (2021). Don’t put all social network sites in one basket: Facebook, Instagram, Twitter, TikTok, and their relations with well-being during the COVID-19 pandemic. PLoS ONE.

[36] Chen S, Shao B, Zhi K (2018). Examining the effects of passive WeChat use in China. International Journal of Human–Computer Interaction.

[37] Thorisdottir IE, Sigurvinsdottir R, Asgeirsdottir BB, Allegrante JP, Sigfusdottir ID (2019). Active and passive social media use and symptoms of anxiety and depressed mood among Icelandic adolescents. Cyberpsychology, Behavior, and Social Networking.

[38] Helm PJ, Jimenez T, Galgali MS, Edwards ME, Vail KE, Arndt J (2022). Divergent effects of social media use on meaning in life via loneliness and existential isolation during the coronavirus pandemic. Journal of Social and Personal Relationships.

[39] Hanley SM, Watt SE, Coventry W (2019). Taking a break: The effect of taking a vacation from Facebook and Instagram on subjective well-being. PLoS ONE.

[40] Valkenburg PM, van Driel II, Beyens I (2021). The associations of active and passive social media use with well-being: A critical scoping review. New Media & Society.

[41] O’Rourke N, Sixsmith A, Study Team BADAS (2021). Ecological momentary assessment of mood and movement with bipolar disorder over time: Participant recruitment and efficacy of study methods. Int J Methods Psychiatr Res.

[42] King DB, O’Rourke N, DeLongis A (2014). Social media recruitment and online data collection: A beginner’s guide and best practices for accessing low-prevalence and hard-to-reach populations. Canadian Psychology/Psychologie canadienne.

[43] Yerushalmi M, Sixsmith A, Pollock Star A, King DB, O'Rourke N (2021). Ecological momentary assessment of bipolar disorder symptoms and partner affect: longitudinal pilot study. JMIR Form Res.

[44] O’Rourke N, Sixsmith A, King DB, Yaghoubi-Shahir H, Canham SL (2016). Development and validation of the BDSx: A brief measure of mood and symptom variability for use with adults with bipolar disorder. Int J Bipolar Disord.

[45] O’Rourke N, Bachner YG, Canham SL, Sixsmith A, Study Team BADAS (2018). Measurement equivalence of the BDSx scale with young and older adults with bipolar disorder. Psychiatry Research.

[46] Osher Y, Bersudsky Y, O'Rourke N, Belotherkovsky D, Bachner YG (2020). Clinical validation of the BDSx scale with bipolar disorder outpatients. Archives of Psychiatric Nursing.

[47] Kraun L, O'Rourke N, Osher Y, Bersudsky Y, Belotherkovsky D, Bachner YG (2020). Is the 6‐item, self‐report HAM‐D an effective depression screening measure with bipolar disorder?. Perspect Psychiatr Care.

[48] Judd LL, Akiskal HS, Schettler PJ, Endicott J, Maser J, Solomon DA, Leon AC, Rice JA, Keller MB (2002). The long-term natural history of the weekly symptomatic status of bipolar I disorder. Arch Gen Psychiatry.

[49] Diener E, Emmons RA, Larsen RJ, Griffin S (1985). The Satisfaction With Life Scale. J Pers Assess.

[50] Diener E (2000). Subjective well-being: The science of happiness and a proposal for a national index. American Psychologist.

[51] Pavot W, Diener E (1993). Review of the Satisfaction With Life Scale. Psychological Assessment.

[52] Pavot W, Diener E, Colvin CR, Sandvik E (1991). Further validation of the Satisfaction With Life Scale: evidence for the cross-method convergence of well-being measures. Journal of Personality Assessment.

[53] O'Rourke N, Heisel MJ, Canham SL, Sixsmith A, Yaghoubi-Shahir H, King DB (2017). Psychometric validation of the Geriatric Suicide Ideation Scale (GSIS) among older adults with bipolar disorder. Aging & Mental Health.

[54] Hays R, DiMatteo MR (1987). A short-form measure of loneliness. J. of Personality Assessment.

[55] Wu C, Yao G (2008). Psychometric analysis of the short-form UCLA Loneliness Scale (ULS-8) in Taiwanese undergraduate students. Personality and Individual Differences.

[56] Zimet GD, Dahlem NW, Zimet SG, Farley GK (1988). The multidimensional scale of perceived social support. Journal of Personality Assessment.

[57] Canty-Mitchell J, Zimet GD (2000). Psychometric properties of the Multidimensional Scale of Perceived Social Support in urban adolescents. Am J Community Psychol.

[58] Zimet G, Powell SS, Farley GK, Werkman S, Berkoff KA (1990). Psychometric characteristics of the Multidimensional Scale of Perceived Social Support. J Pers Assess.

[59] Gerson J, Plagnol AC, Corr PJ (2017). Passive and Active Facebook Use Measure (PAUM): Validation and relationship to the Reinforcement Sensitivity Theory. Personality and Individual Differences.

[60] Verduyn P, Lee DS, Park J, Shablack H, Orvell A, Bayer J, Ybarra O, Jonides J, Kross E (2015). Passive Facebook usage undermines affective well-being: Experimental and longitudinal evidence. Journal of Experimental Psychology: General.

[61] O'Rourke Norm, Hatcher Larry (2013). A Step-by-Step Approach to Using SAS for Factor Analysis and Structural Equation Modeling.

[62] Byrne BM (2016). Structural Equation Modeling With AMOS Basic Concepts, Applications, and Programming, Third Edition.

[63] Cohen J (1989). Statistical Power Analysis for the Behavioral Sciences (2nd ed.). Statistical Power Analysis for the Behavioral Sciences (2nd ed.).

[64] Bauer MS, Simon GE, Ludman E, Unützer J (2018). ‘Bipolarity’ in bipolar disorder: Distribution of manic and depressive symptoms in a treated population. Br J Psychiatry.

[65] Dominiak M, Kaczmarek-Majer K, Antosik-Wójcińska AZ, Opara KR, Olwert A, Radziszewska W, Hryniewicz O, Święcicki ?, Wojnar M, Mierzejewski P (2022). Behavioral and Self-reported Data Collected From Smartphones for the Assessment of Depressive and Manic Symptoms in Patients With Bipolar Disorder: Prospective Observational Study. J Med Internet Res.

[66] Verduyn P, Gugushvili N, Kross E (2021). The impact of social network sites on mental health: distinguishing active from passive use. World Psychiatry.

[67] Harvey D, Lobban F, Rayson P, Warner A, Jones S (2022). Natural language processing methods and bipolar disorder: scoping review. JMIR Ment Health.

[68] Trifiro BM, Gerson J (2019). Social media usage patterns: research note regarding the lack of universal validated measures for active and passive use. Social Media + Society.

[69] van den Eijnden RJ, Lemmens JS, Valkenburg PM (2016). The Social Media Disorder Scale. Computers in Human Behavior.

